# Accounting for uncertainty during a pandemic

**DOI:** 10.1016/j.patter.2021.100310

**Published:** 2021-08-13

**Authors:** Jon Zelner, Julien Riou, Ruth Etzioni, Andrew Gelman

**Affiliations:** 1 Department of Epidemiology, Center of Social Epidemiology & Population Health, University of Michigan School of Public Health, Ann Arbor, MI, USA; 2 Institute of Social and Preventive Medicine, University of Bern, Bern, Switzerland; 3 Fred Hutchinson Cancer Research Center, University of Washington, Seattle, WA, USA; 4 Department of Statistics and Department of Political Science, Columbia University, New York, NY, USA

## Abstract

We discuss several issues of statistical design, data collection, analysis, communication, and decision-making that have arisen in recent and ongoing coronavirus studies, focusing on tools for assessment and propagation of uncertainty. This paper does not purport to be a comprehensive survey of the research literature; rather, we use examples to illustrate statistical points that we think are important.

## The bigger picture

Just as war makes every citizen into an amateur geographer and tactician, a pandemic makes epidemiologists of us all. Instead of maps with colored pins, we have charts of exposure and death counts; people on the street argue about infection fatality rates and herd immunity the way they might have debated wartime strategies and alliances in the past. The severe acute respiratory syndrome coronavirus 2 (SARS-CoV-2) pandemic has brought statistics and uncertainty assessment into public discourse to an extent rarely seen except in election season and the occasional billion-dollar lottery jackpot. In this paper, we reflect on our role as statisticians and epidemiologists and lay out some of the challenges that arise in measuring and communicating our uncertainty about the behavior of a never-before-seen infectious disease. We look at the problem from multiple directions, including the challenges of estimating the case fatality rate (i.e., proportion of individuals who will die from the disease), the rate of transmission from person to person, and even the number of cases circulating in the population at any time. We advocate for an approach that is more transparent about the limitations of statistical and mathematical models as representations of reality and suggest some ways to ensure better representation and communication of uncertainty in future public health emergencies.

## Teaser

Characterizing and communicating uncertainty has been a signal challenge of the COVID-19 pandemic. This uncertainty has touched every aspect of the pandemic, from our understanding of case fatality rates, geographic patterns of infection, and variation in the rate of transmission between people and over time. In this paper, we discuss issues of statistical design, data collection, analysis, communication, and decision-making that have arisen in recent and ongoing studies of SARS-CoV-2, focusing on tools for assessment and propagation of uncertainty.

## Statistics and uncertainty

Just as war makes every citizen into an amateur geographer and tactician, a pandemic makes epidemiologists of us all. Instead of maps with colored pins, we have charts of exposure and death counts; people on the street argue about infection fatality rates and herd immunity the way they might have debated wartime strategies and alliances in the past.

The severe acute respiratory syndrome coronavirus 2 (SARS-CoV-2) pandemic has brought statistics and uncertainty assessment into public discourse to an extent rarely seen except in election season and the occasional billion-dollar lottery jackpot. Statistical claims become political claims and vice versa, with political and ideological positions impacting how we interpret the meaningfulness and uncertainty of statistical results.^1^ As statisticians and epidemiologists, we attempt to contribute to this discourse by laying out some of the challenges that arise in assessing uncertainty and propagating it through statistical analysis and decision-making. We consider several examples and conclude with some general recommendations.

Statistics is key throughout the life cycle of a scientific project, from design through data collection and analysis, and ultimately through communication of results for policy recommendations. In the case of a pandemic, such as SARS-CoV-2, surveillance data are critical for assessment of current status and for future projection, and clinical measurements are vital for evaluating diagnostic tests and intervention efficacy. Design includes sample size calculations, determination of comparison groups and time horizons, and randomization, and is critical in research to identify effective treatments and vaccines. Analysis includes evaluation and estimation based on clinical studies, as well as disease modeling studies, for forecasting and decision support. Communication includes the challenge of drawing inferences and making decisions based on a variety of models and data sources. Uncertainty is present at each step.

## Data and measurement quality

It is becoming painfully apparent that the numbers defining the global burden of SARS-CoV-2 are at best uncertain and at worst completely wrong. The bread and butter of disease surveillance—cases and deaths—are both suspect, for reasons that are only beginning to be fully understood. Studies that rely on these as inputs, for example, for estimating transmission dynamics or case fatality rates, have commonly made the mistake of considering these numbers as a given (and reliable) and do not account for uncertainty or bias in reporting.

### Incidence, prevalence, and mortality

There has been some compelling reporting on how the number of deaths reported in the first few months of the pandemic far exceeds what would have been expected at that time of the year, particularly in states, such as New York, along with analysis of poor alignment between burden and testing.^2^ There has also been good reporting about the confusion arising from differences across states in reporting of COVID-19-related deaths.^3^ Some states have changed how they classify deaths due to COVID-19, leading to potential increases in death counts in some cases (e.g., by including suspected and confirmed SARS-CoV-2 infections in Michigan) and reductions in death counts in others (e.g., Colorado's removal of individuals with COVID-19 at the time of death but for which COVID-19 was not the attributed cause of death from the official COVID-19 death count).^4^^,^^5^

One big question in the early phases of the pandemic was understanding how changes in test availability and distribution both between regions and groups, and over time (for example, as a result of inadequate infrastructure and reagent shortages), impacted our measurements of incidence, prevalence, and mortality, conditional on age and other demographic variables. As the pandemic has worn on and the political and economic costs of high SARS-CoV-2 caseloads have become clear, these issues remain but have shifted from supply considerations to more social ones. For example, political and economic calculations appear to have impacted the accuracy of reporting of nursing home deaths in New York State and may have contributed to a decline in asymptomatic surveillance testing in some states.^6^^,^^7^ Since progress in the pandemic in the US has often been assessed using state-to-state comparisons, this has likely led to erroneous conclusions about what works and what does not, as well as misrepresenting the overall trajectory of the pandemic.

Missing data can also have serious implications for making between-group comparisons. For example, recent work has shown that race/ethnic disparities in COVID-19 incidence and mortality are likely to be dramatically underestimated in complete-case analyses when cases missing race/ethnicity are dropped.^8^ This suggests that the horrific disparities in COVID-19 incidence and mortality are likely even larger than those reported in scholarly research and administrative reports. It could be possible to leverage missingness of key covariates. For example, death certificates typically have more complete information on race/ethnicity than case reports, and a joint model could allow us to efficiently marginalize over these missing covariates, which in preliminary work reveals disparities in mortality that are considerably greater than when these data are dropped. Here, we are using census data to inform the probability that people who are missing race/ethnicity data will be in the mortality versus case-only data.

One way to address data quality is to triangulate. In a clinical study, a hospital can perform antibody tests and RT-PCR RNA tests on patients. In a study tracking symptoms, data can be collected from multiple sources, as in the Carnegie Mellon project, which tracks Facebook and Google surveys, hospital records, web searches, and flu tests.^9^ When measurements cannot be easily calibrated, inferences can be sensitive to assumptions; for example, results of the controversial Stanford antibody study were dependent on assumptions about the sensitivity and specificity of the test.^10^^,^^11^ One additional challenge is the communication of uncertainty in these tests: there is a desire to imagine that the binary test results are conclusive one way or the other instead of essentially representing a probabilistic statement about whether an individual is infected or not.

### Transmission dynamics

These issues are no less pronounced when contemplating population-level transmission dynamics. The basic reproduction number, $R_{0}$, and its cousin the effective reproduction number, R, which measures the actual number of infections generated by an average case, are often cited as measures of inter-human transmissibility and epidemic control. However, it is easy to forget that $R_{0}$and R are not empirical quantities. They are estimated on the basis of surveillance data, which as noted above, is not as reliable as we might wish to believe. In addition, R is a function of (1) the per-contact infectiousness of each individual and (2) the rate at which those contacts occur. Reduce either or both of these and you are likely to reduce the rate of spread. In addition, both measures represent average estimates of a parameter subject to between-individual and temporal variation, due, for example, to variable compliance with social distancing efforts, variation in the extent of viral shedding or age-specific differences in contact and infectiousness. This variation is widely understood in infectious disease epidemiology, and there are theoretical and statistical modeling frameworks that allow us to account for inter-individual variability in susceptibility and infectiousness.

Drivers of variation in infectiousness and susceptibility at an individual or population level can be studied using a hierarchical approach. In this area, there are at least three key dimensions of uncertainty that we need to consider: (1) What range of values of the average infectiousness is consistent with the observed data? (2) How much between-individual variation is there in infectiousness/susceptibility, and how much does it matter to address it specifically? (3) If we implement an intervention to reduce the value of $R_{0}$, how can we estimate how well it worked?

The Imperial College group has fit some reasonable models trying to untangle effects of different policies on the spread of coronavirus, making use of variation in space and time of the growth rates of the infection, and similar issues arise with variation in vaccine uptake.^12^^,^^13^

## Design of clinical trials for treatments and vaccines

Part of designing a study is accounting for uncertainty in effect sizes. Unfortunately there is a tradition in clinical trials of making optimistic assumptions to claim high power. Here is an example that came up in March, 2020. A doctor was designing a trial for an existing drug that he thought could be effective for high-risk coronavirus patients. He contacted one of us to check his sample size calculation: under the assumption that the drug increased survival rate by 25 percentage points, a sample size of N = 126 would assure 80% power. (With 126 people divided evenly in two groups, the standard error of the difference in proportions is bounded above by √(0.5∗0.5/63 + 0.5∗0.5/63) = 0.089, so an effect of 0.25 is at least 2.8 standard errors from zero, which is the condition for 80% power for the z-test.) When we asked the doctor how confident he was in his guessed effect size, he replied that he thought the effect on these patients would be higher and that 25 percentage points was a conservative estimate. At the same time, he recognized that the drug might not work. We asked the doctor if he would be interested in increasing his sample size so he could detect a 10 percentage point increase in survival, for example, but he said that this would not be necessary.

It might seem reasonable to suppose that a drug might not be effective but would have a large individual effect in case of success. But this vision of uncertainty has problems. Suppose, for example, that the survival rate was 30% among the patients who do not receive this new drug and 55% among the treatment group. Then in a population of 1,000 people, it could be that the drug has no effect on the 300 of people who would live either way, no effect on the 450 who would die either way, and it would save the lives of the remaining 250 patients. There are other possibilities consistent with a 25 percentage point benefit—for example, the drug could save 350 people while killing 100—but we will stick with the simple scenario for now. In any case, the point is that the posited benefit of the drug is not "a 25 percentage point benefit" for each patient; rather, it is a benefit on 25% of the patients. And, from that perspective, once we have accepted the idea that the drug works on some people and not others—or in some comorbidity scenarios and not others—we realize that "the treatment effect" in any given study will depend entirely on the patient mix. There is no underlying number representing the effect of the drug. Ideally one would like to know what sorts of patients the treatment would help, but in a clinical trial it is enough to show that there is some clear average effect. Our point is that, if we consider the treatment effect in the context of variation between patients, this can be the first step in a more grounded understanding of effect size.

Many other issues arise when considering clinical trial designs in a pandemic, most notably balancing the goal of reducing uncertainty about the treatment effect and the goal of getting a treatment or vaccine into the population as soon as possible. We recommend that policymakers attempt to quantify the potential risks and benefits of early or late decisions in the design stage, rather than relying on power calculations based on statistical significance.

One issue that arises is what to make of different vaccine efficiency estimates coming from studies conducted at different points in time, in different contexts, and potentially with a differential mix of pathogens floating around? The estimates that are commonly reported refer to symptomatic infection. For the purposes of arresting the toll of mortality in the COVID-19 pandemic, it is most important that vaccines prevent severe disease and death. From this perspective, all the available options do a good job. Arguably this is the number that should be emphasized for the public.

## Disease transmission models

Infectious disease transmission models have been held to unprecedented and deserved scrutiny during the COVID-19 crisis. The field of infectious disease modeling finds its roots in the work of Ross^14^ on malaria, using mathematical tools to describe the complex relations between parasites, vectors, and hosts. Ross defined the concept of dependent happenings, whereby the frequency of an event, such as an infection in an individual, depends on the number of individuals already affected.^15^ Kermack and McKendrick^16^ formalized this approach, leading to the development of the SIR (susceptible-infectious-recovered) differential equation system that is still the basis of many of the models used for SARS-CoV-2 today. In the SIR model, the processes of contagion and immunity are modeled following the mass action principle: the incidence of new infections is dependent on the proportion of infectious and susceptible individuals in the population, assuming homogeneous mixing. In the following decades, the field of infectious disease modeling has seen tremendous development but has long been kept separated from statistical modeling and inference. The focus was on putting theory into equations and exploring different scenarios, leading to important developments in the development and understanding of interventions aimed at controlling epidemics, such as vaccines or vector control. Until recently, comparatively less attention has been given to statistical concepts, such as inference, measurement, and uncertainty.

Several types of approaches have been used to model the transmission of SARS-CoV-2, depending on the stage of the epidemic and the objectives of the work.

Whether the objective of a model is inference, forecasting, or intuition-building, the handling of uncertainty should be a central concern. We can distinguish three sources of uncertainty:

1. *Stochastic uncertainty* arises from chance events during the course of transmission (whether a contact between an infectious and a susceptible person will result in transmission) or data generation (sampling variation in infected individuals that are reported as cases).
2. *Parameter uncertainty* represents the imperfect level of knowledge of a particular quantity, such as the average duration of the incubation period, which is a fixed input parameter to most transmission models.
3. *Model (or structural) uncertainty* refers to the set of assumptions underlying any modeling attempt and their adequacy to reality.^17^ To avoid overconfidence, especially when results are expected to impact policy, one should acknowledge and discuss the potential impact of each of these sources of uncertainty, and as often as possible directly propagate the uncertainty into the results.

### Case example: Estimating transmission rates from early reports

In the early stages of the emergence of SARS-CoV-2 in Wuhan, China, a key focus was estimating the basic reproduction number $R_{0}$ from data on reported cases of SARS-CoV-2 infection. $R_{0}$ is defined as the average number of secondary cases that are generated by an infectious individual in a fully susceptible population. In the first few weeks after its emergence, it was reasonable to assume that the population was fully susceptible to SARS-CoV-2 infection, allowing the use of simple models based on branching processes or exponential growth. Estimating $R_{0}$from counts of reported cases constitutes a typical inference problem and must account for important considerations regarding stochastic, parameter, and model uncertainty.

### Stochastic uncertainty

In the context of emerging pathogens, stochastic uncertainty can be important and, at the stage at which few people are affected, any outlier behavior can have a strong impact on the course of the disease. One key component here is the assumed distribution in the number of secondary cases. In a totally susceptible population, its average is by definition$R_{0}$, but this can vary from individual to individual, with the extreme being a superspreading event (defined as an unusually large number of secondary cases generated by a single infectious person). Superspreading events can have a considerable impact in the early stages of disease emergence by accelerating the spatial spread of the pathogen, as was seen during the emergence of Middle East respiratory syndrome coronavirus.^18^ Two introductions of the same pathogen with the same transmissibility (i.e., with the same $R_{0}$) can result in vastly different epidemic trajectories. Consequently, it would be a mistake to overinterpret differences in case counts across countries or areas as differences in transmissibility, especially when the number of cases is small. Similarly, the uncertainty stemming from low case rates constrains the ability to make informative comparisons across time and space, for instance, to identify the causal impact of specific mitigation measures or environmental drivers, such as temperature or air pollution.^19^ Individual heterogeneity and the potential for superspreading events can be accounted for using a negative binomial distribution for modeling the number of secondary cases.^20^

### Parameter uncertainty

Examining the mechanisms leading to the generation of count data gives insight about the basic assumptions that will explicitly or implicitly be part of any attempt at parameter estimation: (1) an initial zoonotic event led to the infection of a number of humans on a given date; (2) each of these cases generated secondary cases ($R_{0}$ cases on average, with a distribution as discussed above); (3) each of these secondary cases generated cases, with a delay that corresponds to the generation time (the gap between two successive generations of cases, which also is a random variable, not a constant); (4) infected cases will have an incubation period, some of the cases will have symptoms, some of the symptomatic cases will seek care, some of the patients will be tested and diagnosed, some of the diagnosed will be reported to the authorities and counted as a case. From these observations, we understand that is not possible to estimate at the same time $R_{0}$, the date and size of the initial zoonotic event, the incubation period, and the generation time from information about the incidence of SARS-CoV-2, as several combinations of these parameters may lead to the same data. To estimate $R_{0}$, it is therefore necessary to incorporate external information about the other parameters. Here enters parameter uncertainty, as overconfidence about the initial conditions or the generation time could result in both systematic bias in estimation and overconfidence—not enough uncertainty—about the value of $R_{0}$.

### Model uncertainty

Thinking about the mechanisms of data generation brings further considerations about model uncertainty. As of April 2021, much remains unknown about the specific factors, timing and location of the emergence of SARS-CoV-2 at the end of 2019.^21^ Putting aside any political aspect, the early phase of emergence of an unknown pathogen is always a chaotic matter, and modeling the transmission of SARS-CoV-2 and other emerging pathogens requires strong assumptions about how the data were generated. For instance, some authors took the number of reported cases in Wuhan in the first few weeks at face value and directly inferred the rate of exponential growth and thus $R_{0}$, implicitly assuming that the proportion of ascertainment (the proportion of cases that end up in the data) was constant over the period considered.^22^^,^^23^ Other authors made explicit assumptions about the shape of variation of ascertainment with time.^24^ Rather than making assumptions about ascertainment in Wuhan, other authors preferred to use data on national and international cases of SARS-CoV-2 identified in areas still unaffected by the turmoil together with traffic data.^25^, ^26^, ^27^ However, this approach carries other assumptions about the representativity of people who traveled from Wuhan to other places. Differences across estimates based on different assumptions may be referred to as model uncertainty, and this in itself is a good reason to consider multiple approaches to study the same issue.

### Accounting for nonstationarity

Beyond the first few weeks following emergence, it becomes more and more implausible to ignore the impact on transmission of disease-related behavior and the accumulation of protective immunity in the population. Whether the objective is prediction or inference, it is essential to account for how behavior and other factors contributing to transmission—and observation—may change over time. The two broad categories of transmission models typically employed can be adapted to this task, but it increases challenges of model identifiability and interpretability.

*Agent-based models* can be used to simulate the detailed behavior and biology of each individual, going as far as to simulate every vehicle moving in a country.^28^ These models can provide useful insight but are often difficult or impossible to fit to data. In contrast, compartmental models divide the population into different states (susceptible, infectious, and removed for the classical SIR model), without considering any difference among individuals within a state.

*Compartmental models* may be considered within a stochastic or a deterministic framework. The stochastic framework considers the probability of occurrence of each event at each time step and, as hinted by its name, is better suited to handle stochastic uncertainty. The deterministic framework relies upon solving systems of ordinary differential equations (ODEs) and leads to the same average results when the number of infected is sufficiently large. The reduction in computational cost associated with solving ODEs instead of simulating a large number of events is important when the objective is inference.^29^

*Alternative approaches*. A third, hybrid approach was developed by the Institute for Health Metrics and Evaluation (IHME), fitting a Gaussian curve to the shape of the epidemic's mortality trajectory, estimating how restrictions, including social distancing enacted in China impacted the time to and height of the peak, and then extrapolating to other settings on the basis of their accumulating mortality data. The assumption of symmetry in the rise and fall of cases, coupled with the rapid rise in cases and deaths in almost every region, meant that the IHME model predicted a much more rapid decline than other models.^30^^,^^31^ As the virus spread across the US, problems with the model became clear, and the IHME replaced it with a hybrid empirical compartmental approach.

Following this and other failed attempts at prediction, people have mostly given up on forecasting the incidence of COVID-19 beyond a few weeks. While transmission models bring important insights about the general dynamics of an epidemic (e.g., concepts, such as herd immunity, vaccine threshold, and final epidemic size), after a year in it is now more widely understood that the incidence of COVID-19 cases and deaths at a given time and place depends on too many converging factors to allow useful forecasting. These factors range from diversity in the viral population, potential seasonality in transmissibility and contact, to variations in risk perception, care-seeking behavior, and vaccine uptake that can in turn be influenced by age, education, and socio-economic status. To some extent, this represents something of a bright spot, or at least a lesson learned about the limits of models and data as tools for decision-making in a complex, fast-moving situation.

### How can we make better use of models to measure and manage uncertainty?

None of this is an argument against using transmission models to look at potential epidemic trajectories; rather we are arguing for greater transparency and humility in making projections. Examples of how to accomplish this include the following recommendations, summarized in Table 1:

**Table 1:** Summary of the different sources of uncertainty and recommendations on how to address them

Source of uncertainty	Interpretation	Recommendations
Stochastic uncertainty	chance events in data-generating mechanisms	- acknowledge variability at all levels by using appropriate probability distributions - present the entire range of possible predictions arising from the fitted model rather than measures of statistical significance
Parameter uncertainty	imperfect knowledge of influential quantities	- propagate uncertainty from parameters through the results and predictions - make use of Bayesian hierarchical models to partially pool information across individuals, locations, and other units of analysis
Model uncertainty	set of assumptions underlying the model	- maximize transparency with open code and public release of data to allow replication - pre-register modeling assumptions in advance of analysis - compare the inferences and predictions of multiple plausible models rather than searching for the “one true model”

*Model-based predictions* should incorporate stochastic uncertainty by including prediction intervals in addition to point estimates. For time series predictions, visualizations of entire trajectories using tools, such as spaghetti plots, showing the impact of propagating uncertainty throughout the run of a model, should be preferred over simply plotting the intervals over time.

*Parameter uncertainty* should directly be propagated in the results. The quantification of uncertainty in the model outcomes is an integral part of the results and should not be relegated to the side as sensitivity analyses. In this regard, the Bayesian framework with its focus on parameter probability distributions is attractive.

*Model uncertainty* can be handled by carefully considering whether the model structure and all relevant assumptions (even implicit) are adapted to the question as well as using technical tools, such as stacking.^32^ Conducting sensitivity analyses with alternative models is always sensible, but there is only so much than a team can do about its own model. It is advisable to rely on other researchers and experts to provide critical assessment of the model by releasing code and data on an appropriate platform. Model uncertainty is best assessed by the community, and this requires transparency. Code sharing will also bring to academia much-needed good practices for programming, and in the long run build more confidence in the field of infectious disease modeling. Ideally, this process of collective validation would take place before new emergencies occur, in some sort of disaster model pre-registration. Disease transmission models are often not entirely disease specific but rather have defining features that relate to the modes of transmission and immunization. This appears in the profound influence that influenza models and other SEIR-like models had over models applied to the SARS-CoV-2 pandemic.

## Multilevel statistical modeling

So far, we have discussed accounting for uncertainty in research design, data collection, and transmission modeling during epidemics. In addition, data analysis using regression and regression-like models can account for uncertainty and variation using multilevel modeling all the way, and decision-making can be based on costs and benefits estimated using model outputs, and not statistical significance. We have relatively little to say about statistical analysis of this sort because this is one area in which there are readily available tools to handle uncertainty and variation.

We are aware of several SARS-CoV-2 analyses that make use of multilevel models and Bayesian inference. The report by Unwin et al.^12^ is an analysis by the Imperial College group that partially pools across US states, and they have presented similar analyses for Europe.^33^^,^^34^ Zelner et al.^35^ used a multilevel approach to capture age-specific and race-ethnic variation in SARS-CoV-2 mortality in Michigan. A partial list has been collected of SARS-CoV-2 projects using the Bayesian inference engine Stan.^36^ Bayesian analysis can also be performed in the data collection stage, allowing more efficient designs.^37^

A challenging issue with statistical models fit during an ongoing epidemic is dealing with unobserved or partially observed data. Well-designed dynamic models that account for time-varying observation processes can deal with some of these issues, but approaches for fitting stochastic dynamic models to partially observed time series data, such as the partially observed Markov process framework,^38^ are typically more computationally and technologically challenging than more familiar regression-like approaches for fitting deterministic models. As a result, deterministic models have had wide influence, despite their weaknesses and often in situations where demographic stochasticity of the transmission process should be accounted for.

Somewhat ironically, early statistical inferences for epidemic models were actually rooted in a stochastic approach known as the TSIR (time series SIR) model which was originally used to account for time-varying birthrates and demographic stochasticity in models of measles transmission.^39^ An appealing aspect of the TSIR is that it is just a transformation of a regression model and so is accessible to researchers and policymakers with statistical training. Unfortunately, due to the data preparation required to fit them, TSIR models are most useful for the analysis of strongly immunizing infections, such as measles, in which the susceptible population can be accurately reconstructed using data on birthrates and historical measles incidence. As a result, for other infections characterized by different dynamics, more complex and technically challenging approaches, such as the aforementioned partially observed Markov process framework, have become useful.

## Communication

To effectively communicate the results of analyses conducted during the pandemic, what they are meant to accomplish needs to be clear. In the context of the COVID-19 pandemic, this raises the problem of effective scientific communication to the central place it has always belonged. This includes communication of key dimensions of uncertainty in risk.^40^ One of the key challenges here is familiar: How does one impart a gestalt understanding of an interval statistic, such as a confidence or credible interval, to as broad of an audience as possible? van der Bles et al.^41^ provide evidence that people recognize uncertainty when presented as an interval and that communicating this openly does not undermine trust in the numbers or message, with verbal expressions of uncertainty being less effective. Another challenge relates to communication of the different ways in which uncertainty arises and the difficulty of picking one apart from another. For example, what do we do when we cannot disentangle process noise, observation noise, and observation bias? We recommend more emphasis on accurately communicating uncertainty in model inferences and predictions, as discussed by Hullman et al.^42^

Much of the controversy surrounding the multiple transmission models used for prediction and planning could be mitigated by a more pragmatic reframing of what these—and all mathematical and statistical models—are all about. Namely, they distill assumptions and data into inferences for outcomes of interest. Understood this way, they are primarily tools for dimension reduction and exploration, rather than divining rods.

One thing we keep hearing in conversations with state government officials is a concern that people just do not understand when they are at risk. Maps and other visuals can give a realistic and visceral sense of what that risk looks like. Many questions of science communication arise here that relate specifically to the translation of theory into models and models into spoken and written language.^43^ Also relevant when mapping science into decisions is what Blastland et al.^44^ call "evidence communication," where the goal is not to convince or nudge people to act in a particular way but rather to "offer evidence in the round" by conveying estimated quantitative benefits and harms, including numerical uncertainty measures, and anticipating and responding to potential areas of confusions.

Another problem relates to the communication of uncertainty in the structure of the models themselves. We have seen an appetite both from the public and from modelers themselves to find the one true model, with the George Box quote proclaiming that “all models are wrong” (which, like the term "social distancing," we hope never to hear again after this year) tacked on to papers and talks as a fig leaf. But we believe the only way forward is to truly metabolize this argument: What if the challenges and failures of prediction and forecasting in this pandemic are not to be overcome by more elbow grease and ingenuity, but instead require moving the inferential and predictive goalposts to better align with what the available data can tell us?

## Information aggregation and decision-making

In addition to quotidian difficulties of accounting for uncertainty that have occupied statisticians and epidemiologists for hundreds of years, the pandemic setting adds challenges of urgency, novelty, high stakes, and nonstop change.

There has been vigorous debate in the news media, social media, and governments regarding possible future paths of the epidemic and how best to mitigate it. One thing that troubled us in the earliest phases of the pandemic response was the emphasis on rapid analysis of complex, incomplete datasets, followed by rapid publication and extensive media coverage. Rapid response is not inherently problematic, but the conjuring of theoretical frameworks and analytic tools on the fly is unlikely to benefit many more people than the authors of the study. Instead, this makes more sense when you have an existing framework and set of tools that you can apply with minor modifications to incoming data, as was the case with a number of groups enlisted in the earliest days of the pandemic, including IHME as well as Imperial and other groups.

This leads us to wonder whether some kind of disaster model pre-registration is in order for future events, so that the generic behavior of the set of potential tools is well understood before being pressed into services. This could be looser than a clinical trial registration but at least gives the key data inputs and outputs and some characterization of expected behavior under different scenarios. Critically, some type of standardization would give the ability to engineer connections between different types of analyses, so that information on, for example, variable PCR testing across geographic areas and demographic groups, can be easily used to inform estimates of disease incidence and prevalence.

This takes us back to the motivating question behind this essay: How can we adequately account for uncertainty in a pandemic? The question is probably better reframed as: How can we be better *prepared* to address the uncertainty inherent in the response to the next pandemic or another catastrophic, unforeseen—but foreseeable—event. An answer to this question may lie in a reimagining of the tools of epidemiological modeling from something that looks a bit more like the engineering perspective and a bit less like the "pure science" perspective. This entails a move away from analyses as one-off exercises that uncover some permanent—or at least durable—truth, toward a more software-like, continuous improvement conception of the products of statistical analysis.
